# Safety and Efficacy of Vaccines during COVID-19 Pandemic in Patients Treated with Biological Drugs in a Dermatological Setting

**DOI:** 10.3390/healthcare9040401

**Published:** 2021-04-01

**Authors:** Oriana Simonetti, Giulio Rizzetto, Elisa Molinelli, Federico Diotallevi, Giulia Radi, Oscar Cirioni, Marcello Mario D’Errico, Annamaria Offidani

**Affiliations:** 1 Clinic of Dermatology, Department of Clinical and Molecular Sciences, Polytechnic University of Marche, 60121 Ancona, Italy; grizzetto92@hotmail.com (G.R.); molinelli.elisa@gmail.com (E.M.); federico.diotallevi@hotmail.it (F.D.); radigiu1@gmail.com (G.R.); annamaria.offidani@ospedaliriuniti.marche.it (A.O.); 2 Clinic of Infectious Diseases, Department of Biomedical Sciences and Public Health, Polytechnic University of Marche, 60121 Ancona, Italy; o.cirioni@univpm.it; 3 Department of Biomedical Sciences and Public Health, Section of Hygiene, Preventive Medicine and Public Health, Polytechnic University of the Marche, 60121 Ancona, Italy; m.m.derrico@staff.univpm.it

**Keywords:** Covid-19 vaccine, biologic therapy, dermatology, TNF-α inhibitors, Interleukin inhibitors

## Abstract

The BNT162b2 and mRNA-1273 vaccines, consisting of mRNA, have recently become available. The absolute novelty of these vaccines introduces questions about their safety and efficacy, especially in patients who are treated with biological drugs in dermatology. The aim of our review was to provide a broad overview of the current use of all available vaccinations in concomitance with biological therapy and to suggest indications for the new mRNA Covid-19 vaccines. We conducted a narrative review of the literature regarding the indications and safety of the various types of vaccines currently available in dermatological patients treated with biological therapy. The safety and efficacy of administering inactivated vaccines in patients undergoing biological therapy with inhibitors of TNF-α, IL-17, IL-12/23, and IL-4/13 was confirmed. Inactivated vaccines can be administered during therapy with inhibitors of IL-23 and IgE, taking into account that the level of evidence is lower due to the lack of specific studies. Live attenuated vaccines were contraindicated in concomitance with all biological therapies considered, except omalizumab. According to this evidence, we assume that there are currently no contraindications to the administration of the new Covid-19 BNT162b2 and mRNA-1273 vaccines during biological therapy with inhibitors of TNF-α, IL-17, IL-12/23, IL-23, and IL-4/13, since these vaccines are comparable to inactivated ones. For patients with chronic urticaria or allergic asthma treated with omalizumab, we currently recommend caution in using the mRNA Covid-19 vaccines (30 min observation). The only contraindications were a previous history of hypersensitivity to the Covid-19 vaccines themself or to their excipients. In conclusion, further randomized clinical trials are needed to evaluate the efficacy of the antibody response in these patients.

## 1. Introduction

The recent Covid-19 pandemic has radically changed people’s lives and the organization of national healthcare systems. In dermatology, numerous solutions have been proposed to minimize the risk of infection while still providing the best care for patients [1]. The recent introduction of the Covid-19 vaccination may lead to a new phase in the fight against the virus, ensuring greater safety and a progressive return to normal life.

The new BNT162b2 and mRNA-1273 vaccines produced, respectively, by Pfizer/BioNTech and Moderna, are currently available and consist of messenger ribonucleic acid (mRNA) capable of inducing human cells to produce specific antigenic portions (spike proteins) of the SARS-CoV-2 virus [2]. Currently, the BNT162b2 vaccine is being administered in the United States of America, the United Kingdom, and in Europe, while the mRNA-127 vaccine has recently achieved authorization from the European Medicines Agency (EMA). However, the sheer novelty of this method raises questions about its safety and efficacy, especially in patients being treated with biological and immunomodulatory drugs [2].

In dermatology, inhibitors of tumor necrosis factor alpha (TNF-α), interleukin (IL)-17, IL-12/23, IL-23, IL-4/13, and immunoglobulin E (IgE) are widely used. In the literature, numerous studies have evaluated the safety and efficacy of available vaccinations; however there is a lack of a synthesizing work in the dermatological field that includes the most frequent biological therapies. The aim of our review was to provide an up-to-date and comprehensive overview of the latest evidence that can be used in clinical practice to guide the choice of available vaccines. In assessing the evidence produced, we assumed that there were also indications for the new Covid-19 vaccine, although no specific data were yet available at the time of the assessment.

However, the importance of vaccinations in patients undergoing biological therapy derives from an increased infectious risk correlated with inhibitors of TNF-a, IL-17, IL-12/23, IL-23 [3,4,5,6]. For this reason, it is important that patients have received all the vaccines required by the national vaccination plan of the country of residence before starting biologic therapy, as well as pneumococcal and influenza vaccines if other risk factors are present.

Psoriasis patients also suffer from a chronic inflammatory condition [7,8] associated with an increased cardiovascular risk [9], alteration of hepatic lipid metabolism [10], inflammatory bowel diseases, arthritis, osteoporosis, and uveitis [11]. Biological therapies target a specific level of the inflammatory pathway, modifying the progression of the disease. The infectious risk varies depending on the type of biological drug considered.

Specifically, IL-17 inhibitors correlate with upper respiratory tract infections, nasopharyngitis, neutropenia, and mucocutaneous candidiasis [12]. In a systematic review [13], TNF-α inhibitors were also found to be associated with upper respiratory tract infections and flu-like syndrome. Severe infections are very unusual and etanercept appears to be the safest drug in this respect [14]. Anti-IL-23 and -IL-12/23 are most frequently associated with upper respiratory tract infections, influenza, and urinary tract infections [11].

In a recent pooled analysis of 2932 patients with atopic dermatitis [15], dupilumab (anti-IL-4/13) was not associated with an increased risk of infection compared to placebo, and reductions in severe infections and non-herpetic skin infections were also reported. However, a slight increase in the incidence of herpetic infections, albeit not severe, was reported. Finally, omalizumab, an IgE inhibitor, may cause an increased risk of helminth infections, although it has been seen to reduce the clinical manifestations of the rhinovirus and related asthma exacerbations [16].

## 2. Materials and Methods

We conducted a narrative review of the literature regarding the indications and safety of the various types of vaccines currently available in dermatological patients undergoing biological therapy. The bibliographic research was carried out on the PubMed database on 10 December 2020 by seeking combinations of the following keywords: vaccine, anti-TNF-α, anti-IL-17, anti-IL-12/23, anti-IL-23, anti-IL-4/13, adalimumab, infliximab, secukinumab, ixekyzumab, brodalumab, risankizumab, dupilumab, and omalizumab. 

The aim of our review was to provide a broad overview of the current use of vaccinations in concomitance with biological therapy. The recent beginning of the vaccination campaign against Covid-19 led us to evaluate the current state-of-the-art of vaccination, integrating it with the newest data available.

We discarded studies that were not in English and preferred randomized, double-blind, multi-center studies, followed by meta-analyses, observational studies, case series, and case reports. 

## 3. TNF-α inhibitors

TNF-α inhibitors are an important treatment option for moderate-to-severe plaque psoriasis [13]. The evaluation of safety and efficacy in developing an adequate immune response to pneumococcal polysaccharide and inactivated influenza vaccine has been conducted in patients with rheumatoid arthritis and psoriatic arthritis [17,18,19,20,21,22]. Treatment with etanercept, adalimumab and infliximab was shown not to affect the response to the pneumococcal vaccine. Concomitant use of methotrexate (MTX) or disease-modifying antirheumatic drugs (DMARDs) may result in a lower antibody response to the pneumococcal polysaccharide vaccine, although they do not alter the response to the inactivated influenza vaccine [17,23]. These two vaccinations are very important in patients receiving anti-TNF therapy to reduce complications related to those infections.

The administration of live attenuated vaccines is contraindicated in concomitance with TNF-α inhibitors due to the increased infectious risk and lack of available data. However, one study by Suissa et al. [24] showed a possible protective role of etanercept in exacerbations of chronic obstructive pulmonary disease, making it the first choice in patients at particularly high infectious risk or receiving a live attenuated vaccine.

Transplacental passage of IgG occurs in pregnant women on anti-TNF-α therapy during the third trimester and anti-TNF-α IgG has been found up to 6 months after birth in an infant [25]. For this reason, live attenuated vaccines should be avoided for the first six months in infants of mothers treated with TNF-α inhibitors [26], with the exception of certolizumab pegol [27]. In fact, one death from disseminated tuberculosis occurred in an infant exposed to infliximab and vaccinated with Bacillus Calmette–Guérin [28]. TNF-α inhibitors that can cross the blood-placental barrier can also be excreted in breast milk, so breastfeeding should be avoided if therapy cannot be discontinued [29]. The newborn vaccination schedule should be re-evaluated in the event of exposure to anti-TNF-α therapy, possibly by assessing the serum level of the biologic considered prior to administration of a live-attenuated vaccine [30].

### 3.1. Certolizumab

Certolizumab pegol is a pegylated Fab fragment of a monoclonal antibody targeting TNF-α, indicated in moderate-to-severe plaque psoriasis [31]. In the literature, a randomized phase IV trial on 224 subjects confirmed the efficacy and safety of inactivated trivalent influenza and polysaccharide pneumococcal vaccination in patients with rheumatoid arthritis treated with certolizumab pegol, reporting a humoral response comparable to placebo patients. Lower antibody responses were seen in patients treated with anti-TNF-α and concomitant DMARD (MTX) [32]. 

### 3.2. Adalimumab

Adalimumab is a monoclonal antibody that inhibits the action of TNF-a. In the literature, a randomized, multi-center, double-blind clinical trial involving 226 adult subjects with rheumatoid arthritis reported similar antibody responses to trivalent influenza virus vaccine and 23-valent pneumococcal vaccine in subjects treated with adalimumab or placebo [18]. This underlines the efficacy and safety of vaccination with both pneumococcal capsular polysaccharide antigens and inactivated influenza vaccine.

In a study by Burmester et al. [33], 15,132 patients with rheumatoid arthritis treated with adalimumab were analyzed. Of these, 351 patients received influenza vaccination, reporting fewer infection-related adverse effects than unvaccinated subjects.

No data are available on secondary transmission of infection from live vaccines in patients receiving adalimumab but, given the increased risk of infection, administration is not recommended [34]. 

As for pediatric patients, it is recommended to administer adalimumab after having performed the vaccinations required for age. Administration of live and live-attenuated vaccines to children exposed to adalimumab in utero is not recommended until five months after the last administration of adalimumab to the mother during pregnancy [35,36].

## 4. IL-17 inhibitors

The immunogenicity of inactivated vaccines is maintained with IL-17 inhibitors. Although some studies showed that IL-17 deficient mice are unable to produce an adequate immune response [37], in vitro it has been reported that IL-17 alone is unable to induce an immune response; however, B cells with CD4 and T cells lacking IL-17 produced antibodies [38].

### 4.1. Secukinumab 

Secukinumab is an IgG1/κ monoclonal antibody that selectively inhibits IL-17A. Live vaccines should not be given with secukinumab, while inactivated or non-live vaccines, such as flu vaccines, can be used [39]. A study by Frieder et al. [40] on 32 patients with psoriatic arthritis showed that secukinumab does not alter the antibody response to the influenza vaccine. In an open-label, parallel-group, randomized study by Chioato et al. [41], 50 healthy patients treated with 150 mg of secukinumab and placebo following the administration of the meningococcal vaccine C and the inactivated influenza vaccine were able to develop an adequate immune response, confirming the efficacy of these vaccines.

### 4.2. Ixekizumab

Ixekizumab is an IgG4 monoclonal antibody that inhibits interleukin 17A with high specificity. Since no data are available on the response to live vaccines, the administration of live-attenuated vaccines is not recommended. A randomized, open-label, parallel-group study by Gomez et al. [42] on 83 healthy subjects showed that ixekizumab does not impair the humoral response of inactivated vaccines, specifically antipneumococcal (Pneumovax 23) and tetanus toxoid (Boostrix), received two weeks after administration of 160 mg of ixekizumab and concomitantly with the 80 mg dose. Treatment with ixekizumab did not suppress either T-cell-independent or T-cell-dependent immune responses and demonstrated an excellent safety profile [42].

### 4.3. Brodalumab

Brodalumab is an IgG2 monoclonal antibody that binds to the IL-17 receptor, blocking its possible interaction with the various isoforms of IL-17. Before treatment with brodalumab, it is recommended that all vaccinations required by local immunization guidelines be performed, since live vaccines should not be administered during brodalumab therapy [43]. However, there are no data in the literature on response to live or inactivated vaccines.

## 5. IL-12/23 inhibitors

### Ustekinumab

Ustekinumab is an IgG1κ monoclonal antibody that inhibits IL-12/23. The use of live viral or bacterial vaccines during ustekinumab administration is contraindicated, although no specific clinical studies have been conducted. Ustekinumab administration should be discontinued for at least 15 weeks before administering a live vaccine and may be resumed at least two weeks post-vaccination. Inactivated vaccines can be injected and several studies [44,45] demonstrated an effective immune response for pneumococcal polysaccharide, tetanus toxoid, and influenza vaccine. In the prospective study by Doornekamp et al. [44], 15 patients with Crohn’s disease who were treated with ustekinumab and underwent influenza vaccination (Influvac) were evaluated, reporting an antibody response higher than that of healthy controls. In the study by Brodmerkel et al. [45], 60 patients on ustekinumab therapy for several years were vaccinated with both the 23-valent pneumococcal vaccine and tetanus toxoid, reporting superior antibody responses to untreated psoriatic patients.

## 6. IL-23 inhibitors

### Risankizumab

Risankizumab is a humanized monoclonal antibody, consisting of IgG1, a selective inhibitor of IL-23. Live-attenuated vaccinations are contraindicated, and it is necessary to wait 21 weeks after the last administration of the drug before a live-attenuated vaccine. It is therefore recommended that all necessary vaccinations be carried out at least four weeks before starting risankizumab therapy [46]. There are currently no clinical studies that evaluate the efficacy and safety of vaccinations during risankizumab therapy, but inactivated vaccines can be administered.

## 7. IL-4/13 inhibitors

### Dupilumab

Dupilumab is a monoclonal antibody that inhibits IL-4/13. Live attenuated vaccines should not be administered concomitantly with dupilumab as there are no studies ensuring their safety and clinical efficacy. It is therefore recommended that all planned vaccinations be performed before the start of therapy [47].

In a randomized, double-blind, placebo-controlled trial, adult patients with atopic dermatitis were evaluated for immune responses to T-cell dependent vaccines, such as tetanus toxoid, reduced diphtheria toxoid, and acellular pertussis (Tdap) vaccination, and T-cell independent vaccines, such as polysaccharide meningococcal vaccine, all of them inactivated vaccines. Antibody responses were evaluated after 12 weeks of dupilumab 300 mg weekly administration and were similar in dupilumab-treated and placebo-treated patients. No adverse interactions between any of the non-live vaccines and dupilumab were detected in the study. Therefore, patients taking dupilumab may receive concomitant inactivated or non-live vaccines [48].

Since dupilumab inhibits IL-4/13, which is responsible for the type 2 immune response, T-dependent IgG production is not affected. On the other hand, dupilumab shifts T-independent responses, characterized by IgG4 production, towards a type 1 immune response, with IgG1 production and a potential enhancement of the vaccine response [48].

## 8. IgE inhibitors

### Omalizumab

Omalizumab is an IgE inhibitor used for the treatment of chronic idiopathic urticaria, nasal polyposis and asthma. No drug or vaccine interaction studies have been performed, but there are no contraindications to the use of vaccines in patients receiving omalizumab, unless there is a history of previous allergic and/or anaphylactic reactions related to a past vaccination [49].

## 9. Conclusions

Our review of the literature confirmed the safety and efficacy of administering inactivated vaccines in patients undergoing biological therapy with inhibitors of TNF-α, IL-17, IL-12/23, and IL-4/13. Inactivated vaccines can be administered during therapy with inhibitors of IL-23 and IgE according to the respective technical data sheet, but the level of evidence is lower due to the lack of specific studies. We reported in Table 1 all considered vaccines and current indications. Live attenuated vaccines are contraindicated in concomitance with all biological therapies considered, except omalizumab, since an interaction with the Th1 antibody response mechanism of vaccines is not probable [49]. Most of the data available on the safety of vaccines with concomitant anti-TNF-α administration are related to patients suffering from rheumatoid arthritis or other rheumatological diseases. However, the same drugs are also used in dermatology, often at the same or lower dosages, making the evidence produced comparable.

In our opinion, in cases where specific evidence is not available, such as for brodalumab and risankizumab, the indications identified for other drugs that inhibit the same inflammatory pathway can be considered applicable.

Considering the evidence produced in the literature on vaccines currently available, there are no cases in which the administration of the inactivated vaccine has caused an aggravation of the disease, neither for atopic dermatitis, nor for psoriasis, confirming the safety of this type of vaccine. The only vaccines that have been associated with worsening atopic dermatitis or complications such as eczema vaccinatum are live attenuated ones [50], which do not include the new Covid-19 vaccine.

The aim of this review was to provide guidance for vaccination with the new mRNA Covid-19 vaccines, comparing them in some respects to inactivated vaccines. The main similarity consists in the fact that there is no possibility of developing the infection following administration. The safety of mRNA vaccines is guaranteed as mRNA is a non-infectious, non-integrating platform and presents no potential risk of insertional mutagenesis or infection [48]. Since the contraindications to the use of vaccines in patients receiving biological drugs are related to the potential risk of the vaccine developing the disease, both inactivated vaccines and mRNA vaccines do not present this problem, making them practicable even in immunocompromised subjects [51]. The only doubt remains about the actual immune response in these patients.

According to this evidence, we assume that there are currently no contraindications to the administration of the new Covid-19 BNT162b2 and mRNA-1273 vaccines during biological therapy with inhibitors of TNF-α, IL-17, IL-12/23, IL-23, and IL-4/13, since these vaccines are comparable to inactivated ones. There is no Covid-19 infectious risk linked to BNT162b2 and mRNA-1273 vaccines as mRNA encodes only the spike proteins of the virus [52]. 

However, allergic and/or anaphylactic reactions, including two severe ones resolved with epinephrine in the UK, have occurred in patients with histories of severe allergic diathesis (previous anaphylactic shock for foods and drugs, respectively) who received the new vaccine BNT162b2. An excipient of the vaccine, polyethylene glycol (PEG), is thought to be responsible for the allergic reactions, but this has not yet been demonstrated [53,54]. If the role of PEG in allergic reactions is confirmed, it will be possible to test patients with allergic diathesis for sensitization to this substance, and to limit the exclusion of vaccine administration to positives. Since patients treated with omalizumab suffer from chronic urticaria or allergic asthma, we currently recommend caution when administering the mRNA Covid-19 vaccines in these patients due to the possible risk, not related to omalizumab, of allergic reactions, with a 30 min observation period. On the other hand, a previous history of hypersensitivity to the Covid-19 vaccines themselves or to their excipients constitutes a contraindication to their administration [55].

BNTb2162 and mRNA-1273 are being administered in the United States of America, the United Kingdom and Europe, but as this is the first time in history that an mRNA vaccine has been used, studies to assess its safety and efficacy in dermatological patients receiving biological therapy are not yet available. Further randomized clinical trials are needed to evaluate the efficacy of the antibody response in these patients.

## Figures and Tables

**Table 1:** Available vaccines and biologic therapy.

Vaccine	Anti-TNF-a	Anti-IL-17	Anti-IL-12/23	Anti-IL-23	Anti-IL-4/13	Anti-IgE
**Live-attenuated**	Not indicated	Not indicated	Not indicated	Not indicated	Not indicated	Indicated ^¶^
**Inactivated influenza**	Indicated	Indicated	Indicated	Indicated ^¶^	Indicated	Indicated ^¶^
**Pneumococcus** **polysaccharide**	Indicated	Indicated	Indicated	Indicated ^¶^	Indicated	Indicated ^¶^
Other inactivated *	Indicated	Indicated	Indicated	Indicated ^¶^	Indicated	Indicated ^¶^
**Recombinant VZV** **(Shingrix)**	Indicated	Indicated	Indicated	Indicated ^¶^	Indicated	Indicated ^¶^

* Inactivated vaccines: hepatitis A, hepatitis B, diphtheria, Haemophilus influenzae b, Neisseria meningitides, human papillomavirus, influenza, pertussis, *Streptococcus pneumoniae* (conjugated), parenteral poliomyelitis, tetanus toxoid, parenteral typhoid fever, tick-borne encephalitis, trivalent subunit influenza; **live-attenuated vaccines:** oral poliomyelitis, mumps, measles, oral typhoid fever, yellow fever, varicella zoster. **^¶^** Not contraindicated according to the respective technical data sheet, but the level of evidence is lower due to the lack of specific studies about the vaccine.

## Data Availability

The data presented in this study are available in the article.
